# The evolutionary fate of *rpl32* and *rps16* losses in the *Euphorbia schimperi* (Euphorbiaceae) plastome

**DOI:** 10.1038/s41598-021-86820-z

**Published:** 2021-04-02

**Authors:** Aldanah A. Alqahtani, Robert K. Jansen

**Affiliations:** 1 Department of Integrative Biology, University of Texas at Austin, Austin, TX 78712 USA; 2 Department of Biology, Prince Sattam Bin Abdulaziz University, Al-Kharj, 11942 Saudi Arabia; 3 Centre of Excellence in Bionanoscience Research, Department of Biological Sciences, Faculty of Science, King Abdulaziz University, Jeddah, 21589 Saudi Arabia

**Keywords:** Plant sciences, Plant evolution

## Abstract

Gene transfers from mitochondria and plastids to the nucleus are an important process in the evolution of the eukaryotic cell. Plastid (pt) gene losses have been documented in multiple angiosperm lineages and are often associated with functional transfers to the nucleus or substitutions by duplicated nuclear genes targeted to both the plastid and mitochondrion. The plastid genome sequence of *Euphorbia schimperi* was assembled and three major genomic changes were detected, the complete loss of *rpl32* and pseudogenization of *rps16* and *infA*. The nuclear transcriptome of *E. schimperi* was sequenced to investigate the transfer/substitution of the *rpl32* and *rps16* genes to the nucleus. Transfer of plastid-encoded *rpl32* to the nucleus was identified previously in three families of Malpighiales, Rhizophoraceae, Salicaceae and Passifloraceae. An *E. schimperi* transcript of pt SOD-1-RPL32 confirmed that the transfer in Euphorbiaceae is similar to other Malpighiales indicating that it occurred early in the divergence of the order. Ribosomal protein S16 (*rps16*) is encoded in the plastome in most angiosperms but not in Salicaceae and Passifloraceae. Substitution of the *E. schimperi* pt *rps16* was likely due to a duplication of nuclear-encoded mitochondrial-targeted *rps16* resulting in copies dually targeted to the mitochondrion and plastid. Sequences of RPS16-1 and RPS16-2 in the three families of Malpighiales (Salicaceae, Passifloraceae and Euphorbiaceae) have high sequence identity suggesting that the substitution event dates to the early divergence within Malpighiales.

## Introduction

Plastids evolved from endosymbiosis of a cyanobacterium^1^. Since the primary and secondary endosymbiotic events a tremendous number of genes have transferred to the nucleus of the host cell or have been lost entirely from the plastid genome (plastome)^2^.

Most angiosperm plastomes have a highly conserved gene content ranging between 120 and 130 genes out of the approximately 1000–8000 genes that were present in the cyanobacterial ancestor^3^. There are some exceptions in various lineages including losses/transfers of *infA* in most rosids^4, 5^, *rpl33* in some legume lineages^6, 7^, *rpl32* in Salicaceae^8–11^, Rhizophoraceae^10^, Ranunculaceae^12^, Passifloraceae^13, 14^ and Euphorbiaceae^15–17^, *rps16* in various legumes^6, 18–21^, Salicaceae^8, 9, 22^, Passifloraceae^14^ and Euphorbiaceae^15–17, 23–25^, *rpl22* in Fabaceae, Fagaceae, Passifloraceae, and Salicaceae^13, 14, 26, 27^ and *rpl20* in Passifloraceae^14^.

Gene loss from the plastome in angiosperms is an ongoing process^2, 28^. The missing plastid genes carry out important roles and their fate has been explained by two possible mechanisms that have been verified by experimental and/or bioinformatic approaches. They have been either transferred to the nuclear genome such as *rpl32, rpl22, rps7*, *rpoA* and *infA*^4, 10–12, 14, 26, 27, 29^, substituted by a dual targeted nuclear-encoded mitochondrial gene such as *rps16* in *Medicago truncatula* (Fabaceae), *Populus alba* (Salicaceae) and Passifloraceae^14, 22^, or substituted by a nuclear-encoded mitochondrial gene such as *accD* in grasses^30^, *rpl23* in spinach and *Geranium* (Geraniaceae)^31, 32^ and *rpl20* in Passifloraceae^14^.

Numerous nuclear-encoded gene products are required to return to the plastid to maintain the same level of metabolic complexity of the ancestral cyanobacteria^33^. A considerable number of proteins are targeted back to the plastid as pro-proteins, which are inactive proteins that can be converted into an active form that requires a N-terminal extension called a transit peptide^34^. In order for plastid gene transfers to be successful, the gene must gain elements of nuclear expression and acquire a N-terminal transit peptide^2, 35^. Transit peptide acquisition by exon shuffling of an existing nuclear-encoded plastid targeted gene has been identified; for example, in *Populus*, the transit peptide of *rpl32* was acquired by exon shuffling of a duplicated copy of the Cu–Zn superoxide dismutase gene (SOD1)^11^. A novel transit peptide that was acquired by exon shuffling of unknown nuclear-encoded plastid gene has been identified in Ranunculaceae (*Thalictrum* and *Aquilegia*)^12^.

In order for a plastid gene to be successfully substituted by a nuclear-encoded organelle targeted gene, the upstream (N-terminal) portion of mature protein has to be dually targeted to organelles by one of the following mechanisms: alternative transcriptional initiation^36^, alternative translational initiation^37, 38^ or ambiguous targeting information within N-terminal extension sequences^39^. Comparison of the nuclear copy of RPS16 in angiosperms to RPS16 in *E.coli* showed that dual targeting to the organelles occurred without acquiring a N-terminal extension sequence upstream of the mature protein. For instance, in *Populus alba* and *Medicago truncatula*, the RPS16 nuclear copy has gained targeting information within its mature protein without having a N-terminal extension sequence^22^.

Two genes, *rpl32* and *rps16*, have been characterized as being lost in plastomes and either transferred to the nucleus or substituted by a nuclear-encoded mitochondrially-targeted gene in three families of Malpighiales, Rhizophoraceae, Salicaceae and Passifloraceae^10, 11, 14, 22^. The fate of these two missing genes in other families of Malpighiales (Bonnetiaceae, Hypericaceae*,* Clusiaceae, Podostemaceae, Euphorbiaceae, Malpighiaceae, Chrysobalanaceae, Irvingiaceae, Pyllanthaceae, Erythoxylaceae, Linaceae and Violaceae) has not been characterized^15–17, 23–25, 40–46^. Thus, little is known about the extent of the transfer/substitution of these genes in other families of Malpighiales.

Euphorbiaceae include approximately 6745 species organized into 218 genera and four subfamilies^47^. It is one of the largest families of angiosperms and contains at least ten species that exhibit promising anticancer activity^48, 49^. *Euphorbia* contains over 2000 species, making it one of the largest genera of flowering plants^50^. The genus has unique floral features with a specialized inflorescence, the cyathium, and contains latex in its vegetative parts^51^. The primary purpose of this sap is to protect plants from herbivores and it has been used as anti-inflammation, antiangiogenic, antibacterial and to treat cancer^52^. *Euphorbia* is an important component of arid ecosystems because its succulent stems use CAM photosynthesis, which plays an important role in adapting to arid conditions. The diversification of *Euphorbia* is due, at least in part, to the presence of CAM in the succulent stems^53^.

Previous estimates place the origin and time of divergence of *Euphorbia* in Africa roughly 48 million years ago and it subsequently expanded to the Americas through two long distance dispersal events approximately 30 and 25 million years ago^53^. *Euphorbia schimperi* C. Presl belongs to subgenus *Esula* and it grows mainly as a succulent shrub in rocky environments of open savannahs. *Euphorbia schimperi* is a perennial plant reaching heights of 1.2–1.8 m with tiny ephemeral leaves and pencil-like succulent photosynthetic stems (https://inaturalist.ala.org.au/taxa/343121-Euphorbia-schimperi/browse_photos)^54^. The species is distributed in the southern part of the Arabian Peninsula in Saudi Arabia, Yemen, Oman, Socotra as well as east Africa (Ethiopia, Eritrea)^53, 55^. Among the more than 2000 species of *Euphorbia E. schimperi* is especially important because it is known to have anti-breast and brain cancer properties^56^.

Only 21 species of Euphorbiaceae have complete plastome sequences available in NCBI (accessed on Feb 13, 2021) with ten *Euphorbia* species published and most of these are economically important and have some medicinal activities due to the presence of isoprenoids^15–17, 23–25, 46, 57–64^. Using next generation sequencing technologies and de novo assembly, the plastome and nuclear transcriptome of *Euphorbia schimperi* was sequenced*.* The primary objectives are to examine the fate of the two plastid genes, *rpl32* and *rps16*, that have been lost and plot the phylogenetic distribution of these plastid gene losses/transfers/substitutions across the Malpighiales.

## Results

### General features of *Euphorbia schimperi* plastome

The *Euphorbia schimperi* plastome had a length of 159,462 base pairs (bp) with a pair of inverted repeats (IR) of 26,629 bp, which separate the large single copy (LSC, 88,904 bp) and small single copy (SSC, 17,300 bp) regions (Fig. S1, accession number MT900567). Mapping raw reads to the plastome indicated that the average coverage was 1157×. The genome included a total of 128 genes (17 in IR) including 4 rRNAs (all in IR), 30 tRNAs (7 in IR) and 77 protein-coding genes (6 in IR). The plastome of *Euphorbia schimperi* had three putative gene losses, translation initiation factor 1 (*infA)*, ribosomal protein L32 (*rpl32*) and ribosomal protein S16 (*rps16*)*.*

Alignment of the pseudogene of *rps16* of *E. schimperi* with intact *rps16* of *Manihot esculenta* revealed a 5-bp deletion, 10 bp insertion and 27 nucleotide substitutions within exon 2 causing a frameshift (Fig. S2A). A 250 bp deletion, 11 bp insertion and 338 nucleotide substitutions in the intron of *E. schimperi* caused nearly complete loss of the intron and entire loss of exon 1 (Fig. S2B). In rosid plastomes, *infA* is usually located between *rpl36* and *rps8* with length of about 234 bp. The alignment of the *infA* pseudogene of *E. schimperi* with intact *infA* of *Brexia madagascariensis* revealed a 3-bp deletion, 10 bp insertion and 69 nucleotide substitutions causing a frameshift (Fig. S3). Plastid *rpl32*, which is usually located between *ndhF* and *trnL-UAG* and ranges between 150 and 171 bp, was completely missing from the plastome of *E. schimperi*.

### Assembly of *Euphorbia schimperi* transcriptome and quality assessment

The sequenced Illumina libraries yielded 80,916,952 reads. The total reads used, number of assembled contigs and N50 statistics are in Table 1. Mapped read coverage to the assembly using Bowtie2^65^ was 90.26% (73,033,718 reads). BUSCO indicated that the transcriptome assembly covered 87% and 72.3% of conserved single-orthologs of 100 species of eukaryotes (BUSCOs: 303) and 30 species of embryophytes (BUSCOs: 1440), respectively. Amino acid sequences of the candidate ORFs from the *E. schimperi* transcriptome were used to identify pt *rpl32* and *rps16* genes in the nucleus. Statistics of Trinity translated transcriptome assembly are provided in Supplementary Table S1.

**Table 1:** Statistics of Trinity transcriptome assembly.

Total length of sequence	231,194,199 bp
Total number of contigs	311,629
N25	22,235 sequences ≥ 1870 bp
N50	62,276 sequences ≥ 1127 bp
N75	134,642 sequences ≥ 560 bp
Max contig length	14,119 bp
Mean contig length	742 bp
Total GC count	94,624,984 bp
GC%	40.93%

### Identification of gene transfers and substitution to the nucleus

**Figure 1:**
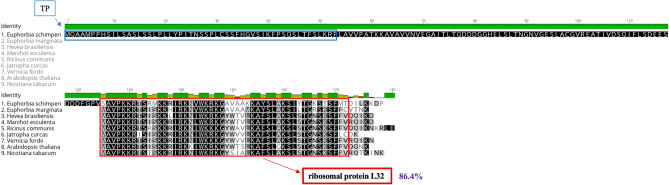
Multiple alignments of nuclear RPL32 of *Euphorbia schimperi* and plastid RPL32 of other Euphorbiaceae (*E. marginata, Jatropha curcas, Hevea brasiliensis, Manihot esculenta, Ricinus communis, Vernicia fordii), Arabidopsis thaliana* and *Nicotiana tabacum.* Blue box indicates plastid transit peptide (TP) predicted using LOCALIZER (~ 53 aa). Red box indicates a conserved domain of RPL32. Pairwise amino acid sequence identity of the conserved domain of nuclear-encoded RPL32 of *E. schimperi* and pt-encoded copies in Euphorbiaceae species and *Arabidopsis thaliana* and *Nicotiana tabacum* was 86.4%.Green histogram indicates amino acid sequence identity.

**Figure 2:**
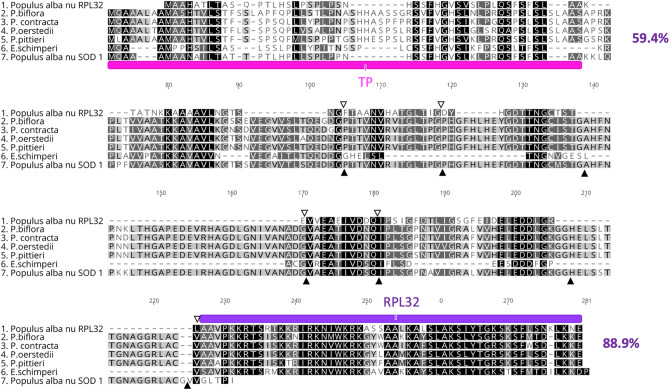
Alignment of the amino acids of nuclear RPL32 of *Euphorbia schimperi, Populus alba* (BAF80584.1)*, Passiflora biflora*; (QKY65178.1)*, P. contracta*; (QKY65180.1)*, P. oerstedii*; (QKY65177.1) and *P. pittieri*; (QKY65179.1) and *Populus alba* nuclear SOD-1(BAF80585.1). Pink annotation indicates plastid transit peptide of SOD-1 in *Populus*. Purple annotation indicates a conserved domain of RPL32 in *Populus*. Open and filled triangles indicate the position of introns in the cp *rpl32* and cp *sod-1* genes in *Populus* (Ueda et al. 2007). Pairwise aa sequence identity of nuclear-encoded RPL32 from *E. schimperi*, *Populus alba*, *Passiflora* (*P*. *biflora, P. contracta, P. oerstedii* and *P. pittieri*) and nuclear-encoded SOD-1 of *Populus alba* was 59.4% and 88.9% for transit peptide and the ribosomal protein L32 conserved domain, respectively. Gaps are indicated by dashes.

Nuclear-encoded RPL32 with high amino acid (aa) sequence identity (82.6%) was detected in the *E. schimperi* transcriptome with upstream sequences of 124 bp from the conserved ribosomal protein L32. Pairwise amino acid sequence identity of the conserved domain of nuclear-encoded RPL32 of *E. schimperi* and pt-encoded copies in Euphorbiaceae species was 86.4% (Fig. 1). The length of nuclear-encoded RPL32 in *E. schimperi* was ~ 179 aa, 55 aa of which represented the conserved domain. This length was similar to the plastid-encoded RPL32 in *Manihot esculenta* (53 aa), *Ricinus communis* (57 aa), *Hevea brasiliensis* (53 aa), *Vernicia fordii* (53 aa), *Jatropha curcas* (50 aa), *Euphorbia marginata* (52 aa), *Arabidopsis thaliana* (52 aa) and *Nicotiana tabacum* (55 aa). TargetP and LOCALIZER analyses of upstream sequences of the ribosomal protein L32 domain strongly predicted a plastid targeted transit peptide (TP) (0.92–1.0). BLAST search (BLASTp) of the TP against NCBI revealed 49–63% aa sequence identity to the cp superoxide dismutase [Cu–Zn] gene (SOD1) of multiple Malpighiales including Euphorbiaceae [*J. curcas* (63%), *M. esculenta* (61%), *H. brasiliensis* (56%) and *R. communis* (49%)], Rhizophoraceae [*Kandelia candel* (52%)] and Salicaceae [*Populus alba* (57%) and *Populus trichocarpa* (55%)]. Pairwise aa sequence identity of nuclear-encoded RPL32 from *E. schimperi*, *Populus alba*, *Passiflora* (*P*. *biflora, P. contracta, P. oerstedii* and *P. pittieri*) and nuclear-encoded SOD-1 of *Populus alba* was 59.4% and 88.9% for transit peptide and the ribosomal protein L32 conserved domain, respectively (Fig. 2).

**Figure 3:**
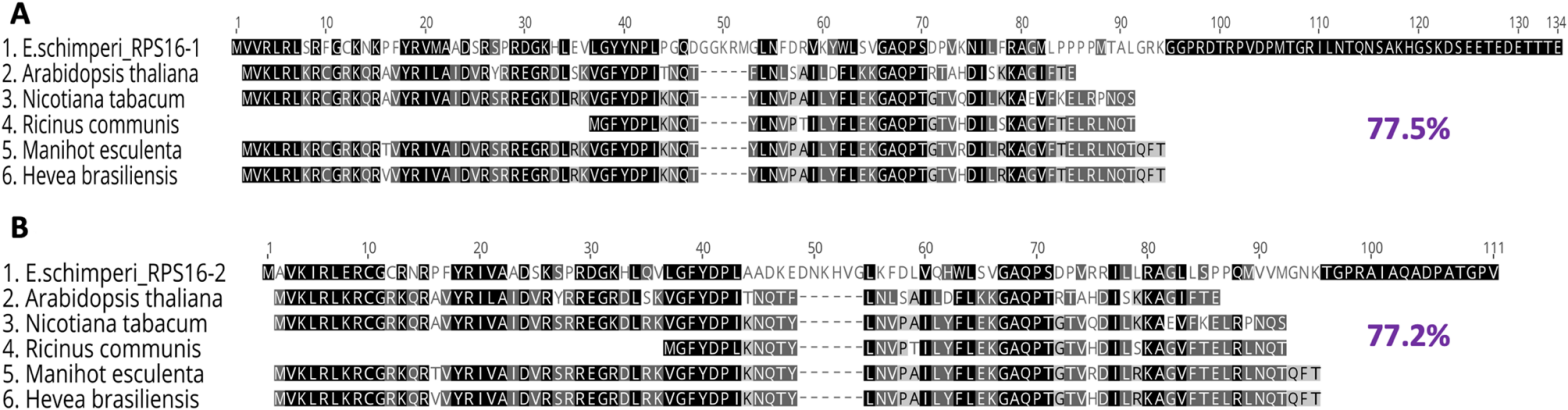
(**A**) Alignments of nuclear RPS16-1 of *Euphorbia schimperi* and plastid RPS16 of other Euphorbiaceae (*Manihot esculenta, Hevea brasiliensis, Ricinus communis), Arabidopsis thaliana* and *Nicotiana tabacum*. (**B**) Alignments of nuclear RPS16-2 of *Euphorbia schimperi* and plastid RPS16 of other Euphorbiaceae (*Manihot esculenta, Hevea brasiliensis, Ricinus communis*)*, Arabidopsis thaliana* and *Nicotiana tabacum*. Pairwise aa sequence identity of the two *E. schimperi* transcripts (RPS16-1 and RPS16-2) with other Euphorbiaceae, *Arabidopsis thaliana* and *Nicotiana tabacum* was 77.5% and 77.2%, respectively. Gaps are indicated by dashes.

**Figure 4:**
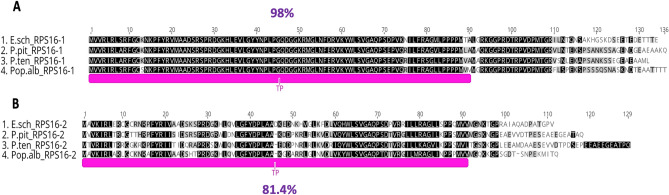
(**A**) Alignment of the amino acid sequences of nuclear RPS16-1 of *Euphorbia schimperi, Populus alba* (RPS16-1: BAG49074.1)*, Passiflora pittieri*; (RPS16-1: QKY65183.1), and P. *tenuiloba*; (RPS16-1: QKY65187.1). (**B**) Alignment of the amino acid sequences of nuclear RPS16-2 of *Euphorbia schimperi, Populus alba* (RPS16-2: BAG49075.1)*, Passiflora pittieri*; (RPS16-2: QKY65185.1), and P. *tenuiloba*; (RPS16-2: QKY65184.1). Pink annotation indicates plastid transit peptide (TP) predicted in *Populus alba* (Ueda et al.^11^). Alignment of the upstream sequences of RPS16-1 and RPS16-2 of *E*. *schimperi* to the transit peptides of RPS16-1 and RPS16-2 of *Passiflora *(*P. pittieri* and *P*. *tenuiloba*) and *Populus alba* resulted in aa identities of 98 and 81.4%, respectively. Gaps are indicated by dashes.

Two transcripts of nuclear-encoded RPS16 were detected in the *E. schimperi* transcriptome. Pairwise aa sequence identity of the two *E. schimperi* transcripts (RPS16-1 and RPS16-2) with other Euphorbiaceae, *Arabidopsis thaliana* and *Nicotiana tabacum* was 77.2% and 77.5% (Fig. 3A,B). The lengths of nuclear-encoded RPS16-1 and RPS16-2 in *E. schimperi* were 134 aa and 111 aa, respectively, and both were longer than the plastid-encoded RPS16 in *M. esculenta* (88 aa), *R. communis* (50 aa), *H. brasiliensis* (88 aa), *Arabidopsis thaliana* (79 aa) and *Nicotiana tabacum* (85 aa) (Fig. 3A,B). Alignment of the upstream sequences of RPS16-1 and RPS16-2 of *E*. *schimperi* to the transit peptides of RPS16-1 and RPS16-2 of *Passiflora pittieri*, *P*. *tenuiloba* and *Populus alba* resulted in aa identities of 98% and 81.4%, respectively (Fig. 4A,B). A BLAST search (BLASTp) of RPS16-1 against NCBI resulted in sequence identity matches with chloroplastic/mitochondrial 30S ribosomal protein S16-1 for multiple species of Malpighiales including Euphorbiaceae [*Manihot esculenta* (80%), *Hevea brasiliensis* (79%), *Jatropha curcas* (63%)], Passifloraceae [*Passiflora tenuiloba* (78.12%), *P. oerstedii* (78%), *P. pittieri* (77.44%)] and other angiosperm lineages, whereas RPS16-2 matched with chloroplastic/mitochondrial 30S ribosomal protein S16-2 of multiple species of Malpighiales including Euphorbiaceae [*Manihot esculenta* (81%), *Hevea brasiliensis* (80%), *Jatropha curcas* (85%)], Passifloraceae [*Passiflora oerstedii* (62%)] and Salicaceae [*Populus trichocarpa* (76%), *P. alba* (76%) and *P. euphratica* (62%)].

A nuclear-encoded copy *of infA* was not found in the transcriptome of *E. schimperi*.

### Phylogenetic distribution of *rpl32* and *rps16* gene losses/transfers/substitutions in Malpighiales

**Figure 5:**
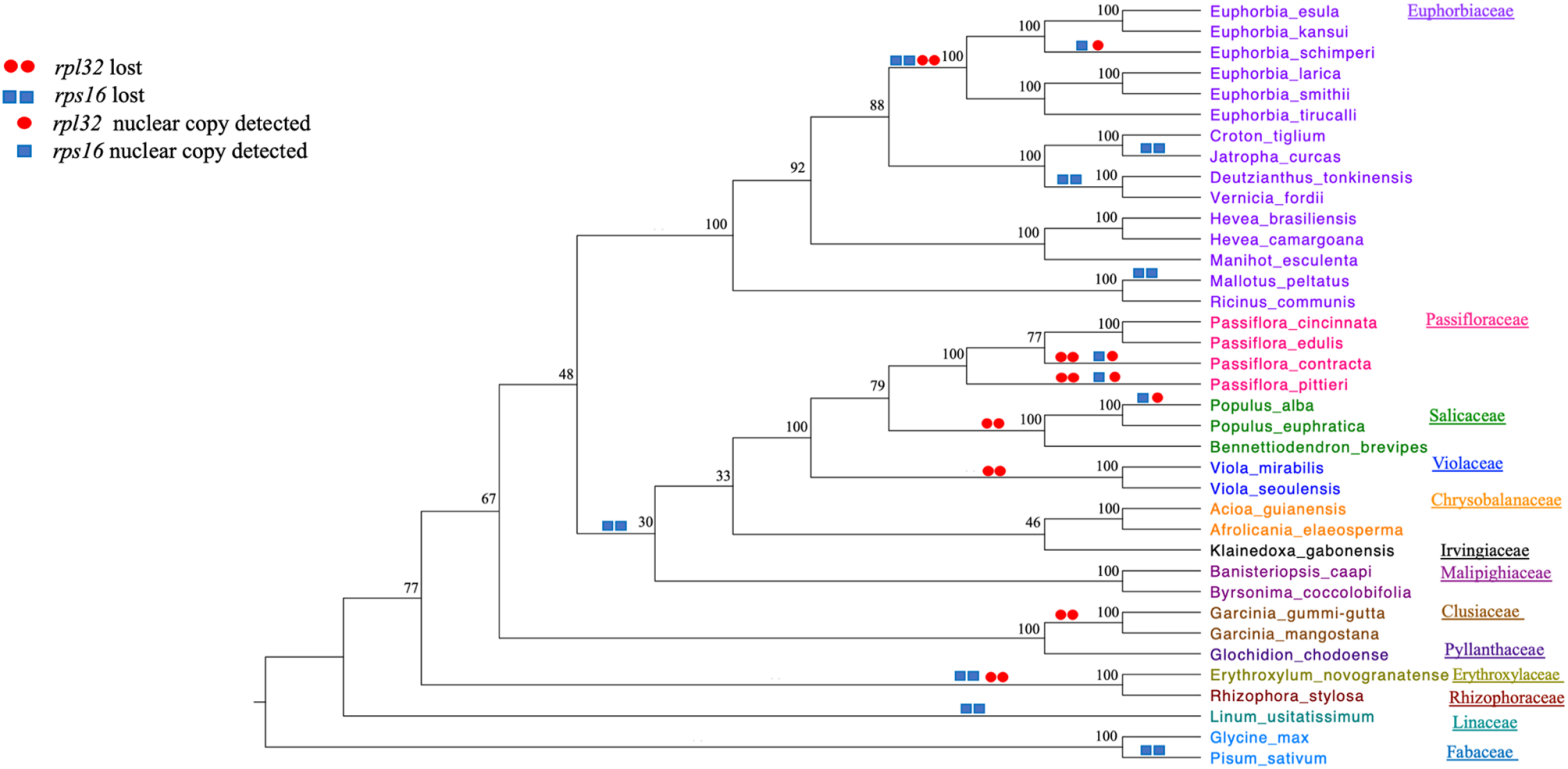
ML cladogram of 37 taxa (Supplemental Table S2) based on 45 plastid gene sequences (Supplemental table S3). Numbers at node are bootstrap values.

The phylogenetic analysis that included 45 protein-coding plastid genes was performed to generate a tree for plotting the distribution of *rpl32* and *rps16* gene losses/transfers or substitutions across Malpighiales (Fig. 5). These changes were plotted based on the results of this study and previously published studies, including examination of the plastome sequences on GenBank (Supplementary Table S2). The results indicated that five genera of Euphorbiaceae have representative species that lost *rps16, Deutzianthus*, *Euphorbia*, *Jatropha*, *Mallotus* and *Vernicia,* and one genus (*Euphorbia*) also lost *rpl32* (Fig. 5)*.* Since all *Euphorbia* plastomes do not have intact *rpl32* or *rps16* these losses likely occurred during the early divergence of the genus. However, the presence of these genes in the nucleus by either a transfer or substitution event has only been documented in *E*. *schimperi*. Some members of other Malpighiales families have experienced the loss of *rpl32* and/or *rps16* from their plastomes, including Passifloraceae, Salicaceae, Violaceae, Erythoxylaceae and Rhizophoraceae, whereas Chrysobalanaceae, Irvingiaceae, Malpighiaceae and Linaceae are missing only *rps16* and Clusiaceae is missing only *rpl32.* The fate of these gene losses has only been determined in *Passiflora* and *Populus* with *rpl32* transferred to the nucleus and *rps16* substituted in selected species in both genera (Fig. 5)^11, 14, 22^.

**Figure 6:**
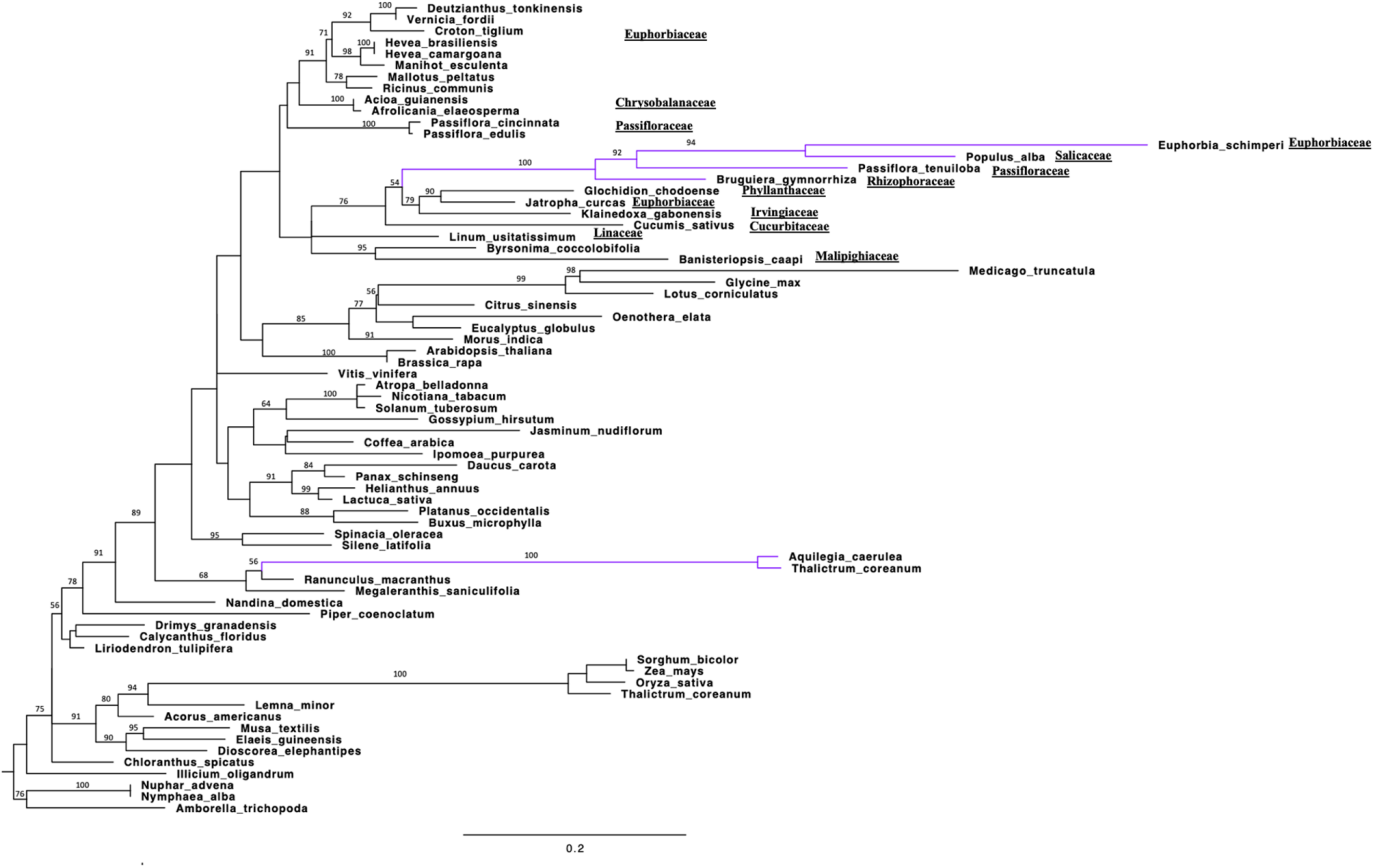
ML phylogram of 71 taxa based on *rpl32* gene sequences. Nuclear copies of *rpl32* are indicated bold purple color. Bootstrap support values > 50% are shown at nodes. Scale bar indicates a phylogenetic distance of 0.2 nucleotide substitutions per site.

Phylogenetic analysis of the second data set included sequences of the *rpl32* gene for 71 species, 65 encoded in the plastid and six in the nucleus. In the resulting phylogram (Fig. 6) the nuclear copy of *E. schimperi* grouped with nuclear copies from the three families Salicaceae (*Populus alba*), Passifloraceae (*Passiflora tenuiloba*) and Rhizophoraceae (*Bruguiera gymnorhiza*). The four species with genes encoded in the nucleus were in a clade of plastid copies from three species in different families of Malpighiales (Euphorbiaceae, Irvingiaceae, Phyllanthaceae) and *Cucurbita* in the Cucurbitales. All other plastid encoded *rpl32* sequences of Malpighiales occurred in a separate clade that was sister to the clade the included the nuclear-encoded copies. Branch lengths of the nuclear-encoded *rpl32* genes from *E. schimperi*, *Populus*, *Passiflora* and *Bruguiera* were much longer than in the plastid-encoded copies.

**Figure 7:**
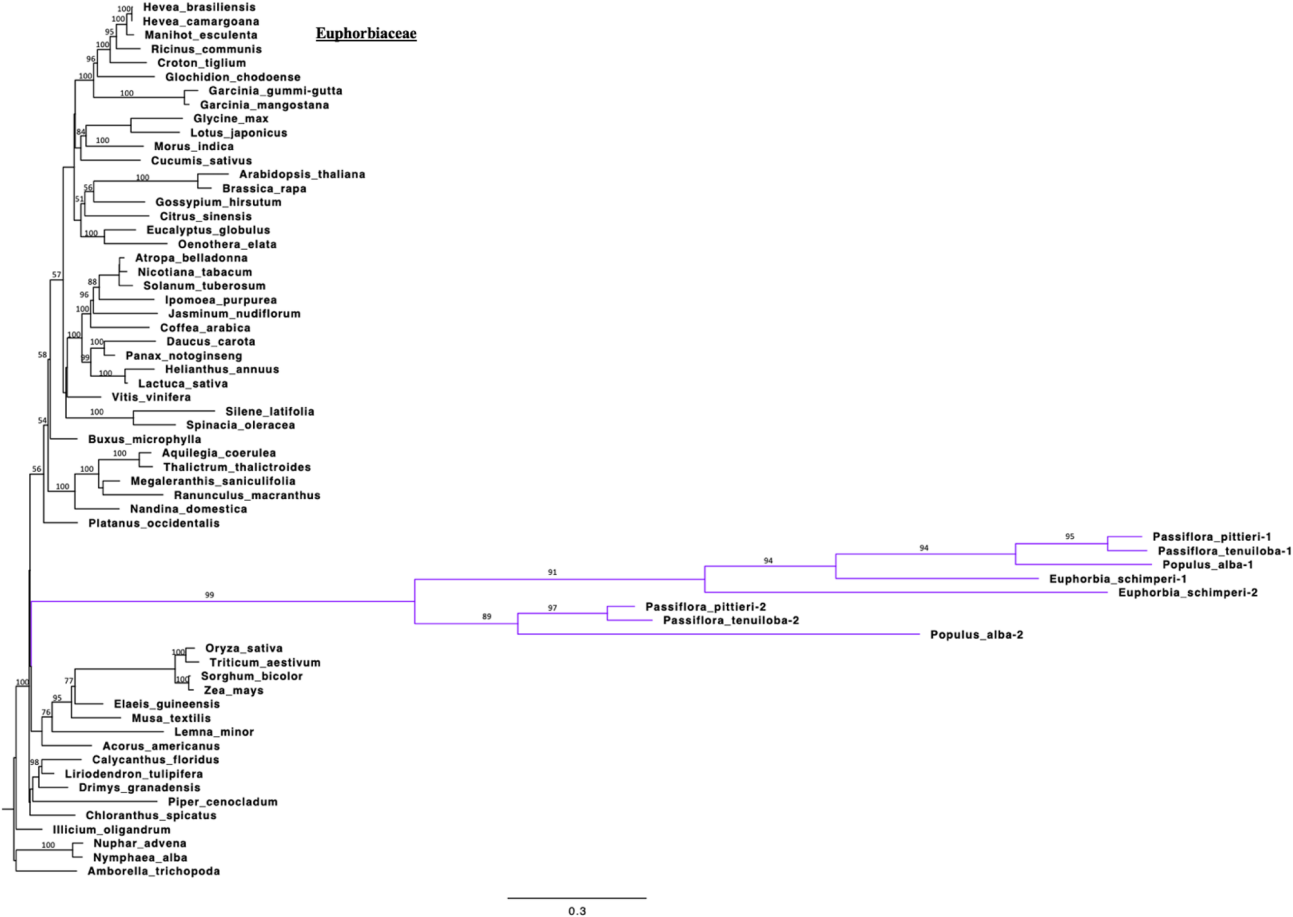
ML phylogram of 60 taxa based on *rps16* gene sequences. Nuclear copies of *rps16* are indicated in bold purple. Bootstrap support values > 50% are shown at nodes. Scale bar indicates a phylogenetic distance of 0.3 nucleotide substitutions per site.

Phylogenetic analysis of the third data set included sequences of the *rps16* gene for 63 species, 55 encoded in the plastid and 8 copies of 4 nuclear genes. In the resulting phylogram (Fig. 7) the nuclear copies of *E. schimperi* grouped with nuclear copies from the two families Salicaceae (*Populus alba*), Passifloraceae (*P. tenuiloba* and *P. pittieri*) in a distant position from plastid encoded copies of Euphorbiaceae. The nuclear-encoded copies resolved as a clade at the base of the eudicots, far removed from plastid-encoded copies of Malpighiales. The long branches of the nuclear-encoded copies of *rpl32* and *rps16* are indicative of the faster substitution rates observed in the nuclear than the plastid genomes^66^. There is some uncertainty in the resolution of the clades due to poor support (bootstrap percentages mostly < 60) among deeper nodes and the artifacts due to long branch attraction.

## Discussion

Pseudogenization or loss of plastid genes is often accompanied by the transfer of the gene to the nuclear genome or substitution by a nuclear gene that is already targeted to the plastid^27, 67^. Plastid gene loss and transfer to the nucleus or substitution by a dual targeted nuclear gene targeted to organelles has been recorded for several genes across multiple angiosperm lineages^4–17^. The focus of this study was to use transcriptome data to bioinformatically identify the fate of two plastid genes, *rpl32* and *rps16*, in *Euphorbia schimperi* that have either been lost or pseudogenized. Plastome sequences of Malpighiales have documented the loss of *rpl32* and *rps16* but the fate of these two genes in most families has not been examined. In this study, the phylogenetic distribution of the loss/transfer/substitution of these genes across the Malpighiales was examined.

The transfer of plastid-encoded *rpl32* to the nucleus has been identified previously in three families of Malpighiales, namely Rhizophoraceae, Salicaceae and Passifloraceae^10, 11, 14^. Cusack and Wolfe^10^ identified the duplication of a nuclear chimeric gene (pt SOD-1-RPL32 fusion protein and pt SOD-1 protein) that is associated with the transfer of *rpl32* in *Populus*. Ueda et al.^11^ experimentally confirmed the functional transfer of the *rpl32* gene from the plastid to the nucleus and showed that the pt SOD-1-RPL32 fusion protein is targeted to the plastid of *Populus* by using green fluorescent protein (GFP). Shrestha et al.^14^ identified high sequence identity of the pt SOD-1-RPL32 fusion protein to pt SOD-1-RPL32 transcript of *Populus* by mapping to the transcriptome of *Passiflora*. In the current study, *Euphorbia schimperi* and other *Euphorbia* species available at NCBI have experienced loss of the *rpl32* gene but no previous studies have been performed to determine the fate of this gene. An *E. schimperi* transcript that represents pt SOD-1-RPL32 has been identified confirming that the transfer in Euphorbiaceae is similar to three other families (Rhizophoraceae, Salicaceae and Passifloraceae) of Malpighiales. Since these four families share a high sequence similarity of the transit peptide derived from pt *sod-1* and the loss from the plastome is widespread in order, the timing of the transfer event may date to the early divergence of this clade (Fig. 5). The other families of Malpighiales have not been examined but they may also have nuclear encoded copies of *rpl32*. If this is the case, the plastid-encoded copies in some Malpighiales may not have been pseudogenized or lost yet in these families. Additional sampling of transcriptomes of other families of Malpighiales is needed to more accurately determine the timing of the *rpl32* transfer to the nucleus. Ranunculaceae (*Thalictrum coreanum* and *Aquilegia caerulea*) experienced an independent transfer of plastid-encoded *rpl32* to the nucleus because its transit peptide sequence is substantially different from the distantly related families of Malpighiales (Fig. 6)^12^.

The substitution of the plastid-encoded *rps16* by a dual targeted nuclear-encoded mitochondrial gene was identified previously in two families of Malpighiales, Salicaceae and Passifloraceae^14, 22^. In *Populus alba* (Salicaceae), Ueda et al.^22^ experimentally localized the two nuclear-encoded *rps16* genes that are dually targeted to the plastid and mitochondrion using GFP. A similar substitution occurs in the monocot *Oryza sativa* and eudicot *Arabidopsis thaliana*^22^*.* In addition, bioinformatic comparisons in Passifloraceae identified RPS16-1 and RPS16-2 in the transcriptome, with one targeted to the plastid and the other to the mitochondrion. *Euphorbia schimperi* plastome sequences and other *Euphorbia* species available at NCBI have experienced pseudogenization of *rps16* gene and no previous studies have elucidated the fate of plastid-encoded *rps16* loss in the genus. In Euphorbiaceae three species of subfamily Crotonoideae, *Jatropha carcus*^23^, *Deutzianthus tonkinensis*^25^ and *Vernicia fordii*^24^, are missing plastid-encoded *rps16*. In most cases the loss of *rps16* is associated with a deletion in the intergenic spacer between *trnK-UUU* and *trnQ-UUG* or an inversion in the same region^23^. The loss of *rps16* in the gymnosperm *Keteleeria davidiana*, the monocot *Dioscorea elephantipes* and eudicots (*Aethionema cordifolium*, *Aethionema grandiflorum*, *Arabis hirsuta*, *Draba nemorosa*, *Lobularia maritima*, *Populus alba*, *Populus trichocarpa*, *Cuscuta gronovii*, *Cuscuta exaltata* and *Epifagus virginana*) is also the result of deletion in the same region^23^. In *Jatropha curcas* the loss of *rps16* gene is associated with a 1.3 kb deletion in the *trnK-trnQ* intergenic region^23^. Likewise, *Euphorbia schimperi* has a deletion of 0.5 kb in the same intergenic region (Fig. S2A,B).

The situation in *Euphorbia schimperi* is similar to Salicaceae and Passifloraceae with two copies, RPS16-1 and RPS16-2, in the nucleus (Fig. 7). Since the three families (Salicaceae, Passifloraceae and Euphorbiaceae) of Malpighiales share a high sequence identity of the RPS16-1 and RPS16-2, the gene substitution likely occurred early in the divergence of Malpighiales (Fig. 5), although comparisons of plastomes and transcriptomes of other families in the order are needed to confirm the timing. Some taxa in these families still retain an intact copy of *rps16* in the plastome but it is not known if these are functional or if they simply have not been lost or pseudogenized yet.

## Conclusion

The sequence of *Euphorbia schimperi* expands the understanding of the evolution of plastomes within Malpighiales. Gene order of *E. schimperi* is highly conserved with the typical structure of the angiosperm plastomes. The only unusual feature of the *E. schimperi* are gene-content changes with the loss of *rpl32*, *rps16* and *infA*. Screening the nuclear transcriptome of *E. schimperi* shows that two of these genes have been either transferred to the nucleus or substituted by a duplicated nuclear-encoded mitochondrially-targeted gene*.* The fate of *infA* was not determined because a nuclear-encoded copy was not found in the transcriptome. Comparisons of the nuclear copies of *rpl32* and *rps16* genes of *E. schimperi* to members of other families of Malpighiales (Salicaceae and Passifloraceae) suggest that the transfer or substitution events in Euphorbiaceae may have occurred early in the divergence of this order.

## Materials and methods

### Plant material and DNA and RNA isolation

*Euphorbia schimperi* plants were obtained from the Arid Land Greenhouses (https://aridlandswholesale.com/) in Tucson, Arizona and grown in the greenhouse at the University of Texas at Austin. A voucher specimen was deposited in the TEX/LL herbarium as Alqahtani s.n. (TEX 00501952). Leaves were harvested from a single plant, flash frozen in liquid nitrogen and stored at -80 C^o^ until isolations were performed. Whole genomic DNA was extracted from 0.2 g of the leaves using the Doyle and Doyle^68^ protocol with the following modifications: 2% PVP and 2% β-mercaptoethanol (Sigma, St. Louis, MO, USA) were added to the cetyl trimethylammonium bromide (CTAB) extraction buffer. The clear aqueous fraction was obtained after repeated separations with chloroform: isoamyl alcohol, followed by precipitation with isopropanol and 3 M sodium acetate. The pellet was washed with 70% ethanol and then resuspended in ~ 200 μl DNase-free water. The sample was subjected to RNase treatment followed by another phase separation with chloroform: isoamyl alcohol and recovered by precipitation with isopropanol and 3 M sodium acetate. The DNA pellet was washed with 70% ethanol, resuspended in ~ 50 μL H_2_O and stored at − 20 °C.

RNA was extracted from 0.25 g of leaves from a single plant that was from the same clone used for the DNA extraction using the RNeasy Plant Mini Kit following the manufacturer’s instructions (Qiagen, Germantown, MD, USA). Using DNase digestion, RNA was treated to eliminate any remaining DNA based on the enzyme protocol (Fermentas #EN0521, 1 unit/μL, Waltham, MA, USA). The 50 μL RNA sample was combined with 30 μL of 10× buffer and 20 μL DNase enzyme for a total volume of 100 μL. After incubation for 1 h at 37 °C DNase was removed using microcolumns and then cleaned with RNA Clean & Concentrator-25 following the manufacturer’s instructions (Zymo Research, Irvine, CA, USA). The quality and quantity of the RNA sample was evaluated with the targeted optimal values of > 200 ng/µL, 260/280 ratio from 1.9 to 2.1, 260/230 ratio between 2.0 to 2.5 and RNA integrity number (RIN) > 8.0.

### Genome sequencing, assembly and annotation

DNA with a concentration of 100 ng/µL and volume of 42 µL was submitted for paired end sequencing (2 × 150 bp) on the Illumina HiSeq 4000 platform at the Genome Sequencing Analysis Facility (GSAF) at the University of Texas at Austin. Velvet v.1.2.07^69^ with multiple K-mer values between 81 to 109 and coverage cutoffs of 200X, 500X and 1000X was used for de novo assembly of the Illumina reads at the Texas Advanced Computing Center (TACC, http://www.tacc.utexas.edu). The resulting contigs from 15 different k-mer parameters assembled with 500X and 1000X coverage were imported into Geneious (v.10.0.6; http://www.geneious.com)^70^. De novo assembly with default settings was run to generate long contigs that represented the entire plastome. Putative plastid contigs were identified using the *Euphorbia esula* plastome as a reference in Geneious. A gap between *ycf1* and *ndhF* was filled by overlapping contigs. The second gap between *atpH* and *atpF* and ambiguous nucleotides were filled by mapping contigs against the raw reads using Bowtie 2 version 2.3.4^65^.

Annotation of the plastome was conducted using multiple software platforms. Geneious was used to check for start and stop codons for every gene compared to *Nicotiana tabacum* (NC_001879.2) and species of Euphorbiaceae available publicly, including *Jatropha curcas* (NC_012224.1)*, Euphorbia esula* (NC_033910.1)*, Manihot esculenta* (NC_010433.1)*, Ricinus communis* (NC_016736.1) and *Hevea brasiliensis* (NC_015308.1)*.* Dual Organellar Genome Annotator (DOGMA) was utilized to identify coding sequences with default settings^71^. The tRNAscan-SE online search server was used to confirm tRNA genes^72, 73^. Based on the loss of portions of the sequence or presence of internal stop codons, pseudogenes were identified. A genome map was drawn using OGDRAW^74^.

### Transcriptome de novo assembly

Standard RNA-Seq with ribosomal RNA removal library preparation and sequencing via Illumina HiSeq 4000 were carried out at GSAF. The quality of raw FastQC reads was examined using the FastQC tool v.0.11.5 (http://www.bioinformatics.babraham.ac.uk/projects/fastqc/)^75^. Raw RNA-seq data was not subjected to quality trimming. A de novo assembly of RNA-seq reads into transcripts was performed using Trinity^76^ with 25 k-mer size^77^. Trinity sequentially integrates Inchworm, Chrysalis and Butterfly modules to process a large number of RNA-Seq reads. This has been used to partition the sequence data into different individual de Bruijn graphs, which represent the transcriptional complexity at a given gene or locus^77^.

### Transcriptome quality assessment and annotation

To validate the de novo assembly, read remapping was conducted using two software packages, Bowtie2 v.2.3.2^65^ and Benchmarking Universal Single-copy Orthologs (BUSCO) v.3.0.2^78^. Bowtie2 index was created for the data and the number of reads that mapped to the transcriptome was counted. BUSCO was carried out using the Embryophyta and Eukaryota databases. BUSCO assessment provided quantitative measures to identify the completeness of the transcriptome based on evolutionarily informed expectations of the gene content from near-universal single-copy orthologs selected from OrthoDB v.9^78^. In addition, N25, N50 and N75 contigs of transcriptome and translated transcriptome were identified. De novo assemblies contain no information about what genes the contigs may correspond to, so the final transcriptome assembly for *E. schimperi* was annotated to identify genes and functional terms the contigs likely correspond to using the BLASTx searches against the protein database (SwissProt) (http://www.uniprot.org) and the predicted *Arabidopsis thaliana* proteome (Tair v.10, http://arabidopsis.org) using the BLAST settings (BLASTx, report 1 hit, e-value of 1e^−5^). TACC was used to conduct the analyses of transcriptome assembly and quality assessments.

### Identification of gene transfer and substitution

The final assembly set of transcripts of *E. schimperi* was subjected to TransDecoder v3.0.1 (https://transdecoder.github.io/) to determine potential coding regions. LongOrfs was used to select the best single open reading frame (ORF) per transcript longer than 100 amino acids. Plastid gene transfer/substitution in *E. schimperi* to the nucleus was examined by performing BLASTp searches of plastid-encoded RPL32 sequences of *M. esculenta* (ABV66201.1), *R. communis* (AEJ82604.1)*, H. brasiliensis* (YP_004327709.1), *V. fordii* (YP_009371112.1)*, J. curcas* (ACN72738.1)*, E. marginata* (AMC32178.1), *Arabidopsis thaliana* (NP_051107.1) and *Nicotiana tabacum* (CAA77431.1) and plastid-encoded RPS16 sequences of *A. thaliana* (NP_051041.1), *N. tabacum* (NP_054479.1), *M. esculenta* (ABV66136.1), *R. communis* (AEJ82537.1) and *H. brasiliensis* (ADO33539.1) against the peptide sequences for the final candidate ORFs of the *E. schimperi* transcriptome*.*

BLASTp searches of the query sequences [nuclear- encoded RPL32 of *Passiflora* (*P. pittieri* (QKY65179.1), *P. contracta* (QKY65180.1), *P. oerstedii* (QKY65177.1) and *P. biflora* (QKY65178.1)], *Populus alba* (BAF80584.1)*, Populus alba* SOD-1 (BAF80585.1) and nuclear- encoded RPS16-1& RPS16-2 copies sequences of *Passiflora* [*P. pittieri* (RPS16-1: QKY65183.1, RPS16-2: QKY65185.1), *P. tenuiloba* (RPS16-1: QKY65187.1, RPS16-2: QKY65184.1)] and *Populus alba* (RPS16-1: BAG49074.1, RPS16-2: BAG49075.1) against the peptide sequences for the final candidate ORFs of *E. schimperi* transcriptome were conducted. BLASTp commands utilized were e-value 1e^-2^ -outfmt 6 -num_threads 4. The query sequences were downloaded from GenBank (https://ncbi.nlm.nih.gov). Nuclear and plastid copies of RPL32 and RPS16 sequences were aligned with MAFFT v7.388^79^ in Geneious.

Putative transit peptides and a mitochondrial targeting peptide of nuclear transferred genes were identified using TargetP-1.1^80^(http://www.cbs.dtu.dk/services/TargetP-1.1/index.php) and LOCALIZER^81^ (http://localizer.csiro.au/). To detect the source of the transit peptide for the nuclear-encoded RPL32 and RPS16, BLAST searches (BLASTp) were conducted against the NCBI database.

### Phylogenetic analyses

Phylogenetic analyses were performed on three data sets. The first included 45 plastid-encoded gene sequences extracted from 35 taxa of Malpighiales and two outgroups in Fabales, *Pisum sativum* and *Glycine max* (Tables S2, S3). The second included both nuclear- (6) and plastid-encoded (65) *rpl32* genes (Table S4). This data set was constructed by adding nine Euphorbiaceae species and ten species of Malpighiales to data (52 species) from Park et al. (2015), which is available in Dryad Digital Repository (http://dx.doi.org/10.5061/dryad.g84g5/Align_52rpl32only_tree). The third included both nuclear-(8) and plastid-encoded (52) *rps16* genes (Table S5). All alignments were performed using MAFFT v.7.388^79^ with default settings in Geneious. Phylogenetic analyses of first data set was conducted using maximum likelihood (ML) in RAxML-NG v.0.9 with the GAMMA GTR model under rapid bootstrapping algorithm with 100 bootstrap replicates (https://raxml-ng.vital-it.ch/#/)^82^. The single gene trees were generated using maximum likelihood (ML) in IQ-TREE v.1.6.12 with the TVM + F + G4 best fit model in *rpl32* gene and GTR + F + I + G4 in *rps16* under rapid bootstrapping algorithm with 1000 bootstrap replicates^83^. FigTree v.1.4.4^84^ was used to visualize phylogenetic trees.

## Supplementary Information

Supplementary Figures.

Supplementary Tables.
