# Supplementary material for: The evolutionary fate of rpl32 and rps16 losses in the Euphorbia schimperi (Euphorbiaceae) plastome
Source: Sci Rep. 2021 Apr 2;11:7466. doi: 10.1038/s41598-021-86820-z (PMC8018952; doi:10.1038/s41598-021-86820-z)
Supplement: Supplementary file 1 — Supplementary Figures. [file 41598_2021_86820_MOESM1_ESM.docx]

**The evolutionary fate of *rpl32* and *rps16* losses in the *Euphorbia schimperi* ( Euphorbiaceae) plastome**

**Aldanah A Alqahtani^1,2^, Robert K. Jansen^1,3^**

^1^Department of Integrative Biology, University of Texas at Austin, TX, 78712, USA

^2^Department of Biology, Prince Sattam Bin Abdulaziz University, Al-Kharj 11942, Saudi Arabia

^3^Centre of Excellence in Bionanoscience Research, Department of Biological Sciences, Faculty of Science, King Abdulaziz University, Jeddah 21589, Saudi Arabia

^*^Corresponding author

Aldanah A. Alqahtani

[aldanaha2016@utexas.edu](mailto:aldanaha2016@utexas.edu)

[Ald.alqahtani@psau.edu.sa](mailto:Ald.alqahtani@psau.edu.sa)


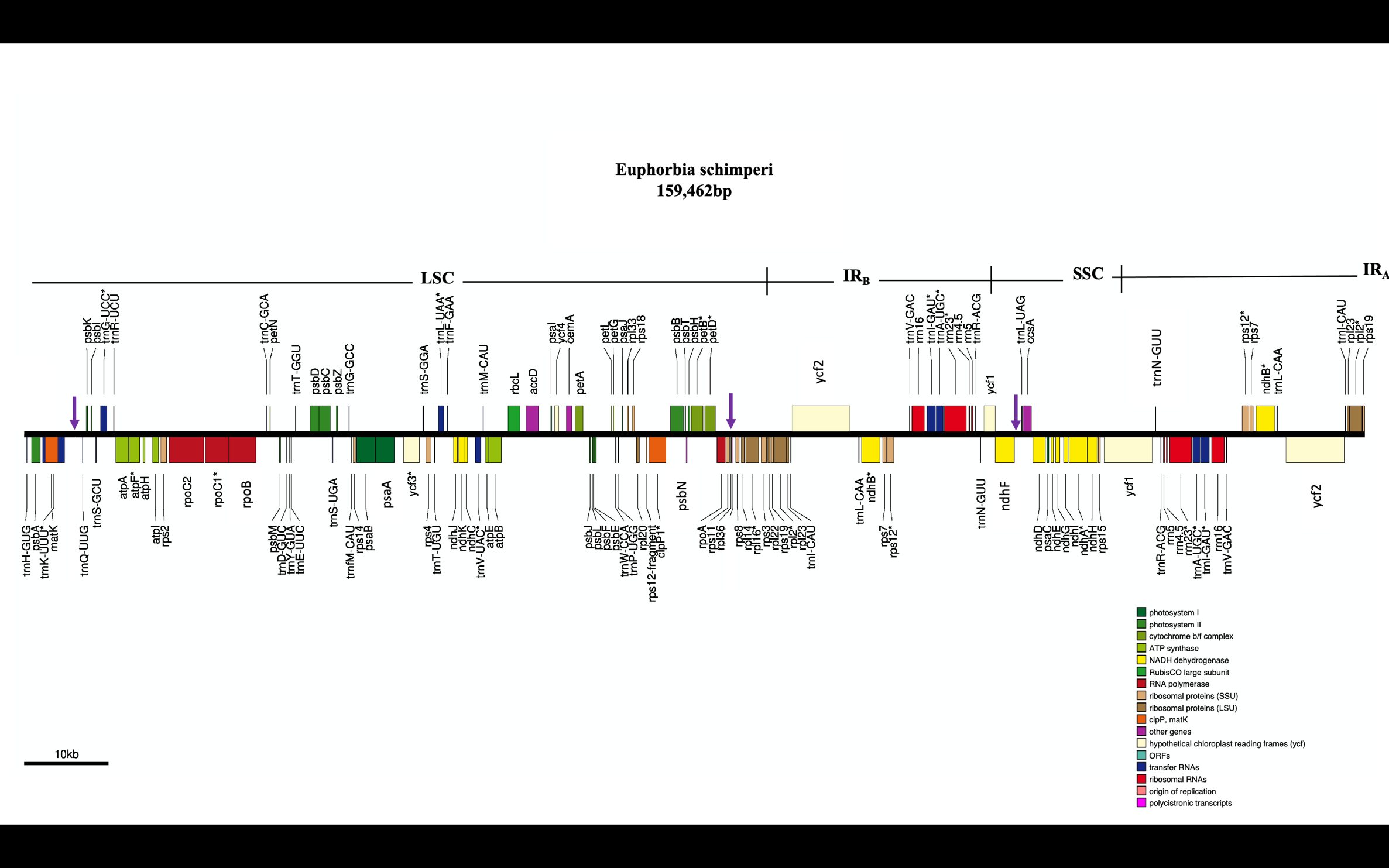
­­

**Figure S1:** Linear map of the *E. schimperi* plastome*.* Thicker lines on map indicate the extent of inverted repeats (IRa, IRb, 26,629 bp) that separate the genome into small single copy (SSC, 17,300 bp) and large single copy (LSC, 88,904 bp) regions. The purple arrows indicate the missing genes where the first arrow from the left indicates *rps16* which is located between *trnK-UUU* and *trnQ-UUG*, *infA* is located between *rpl36* and *rps8* and *rpl32* is located between *ndhF* and *trnL-UAG*.


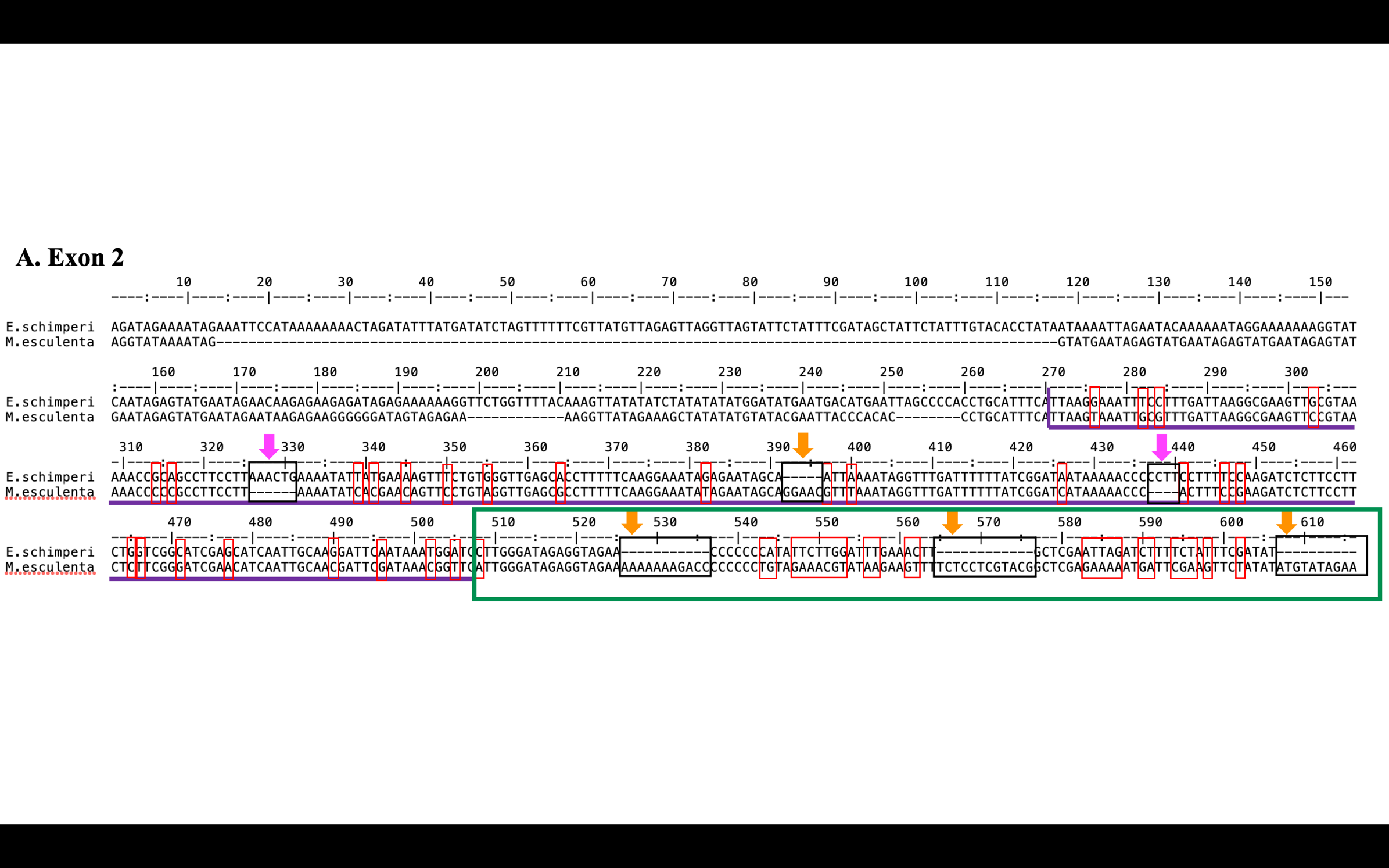


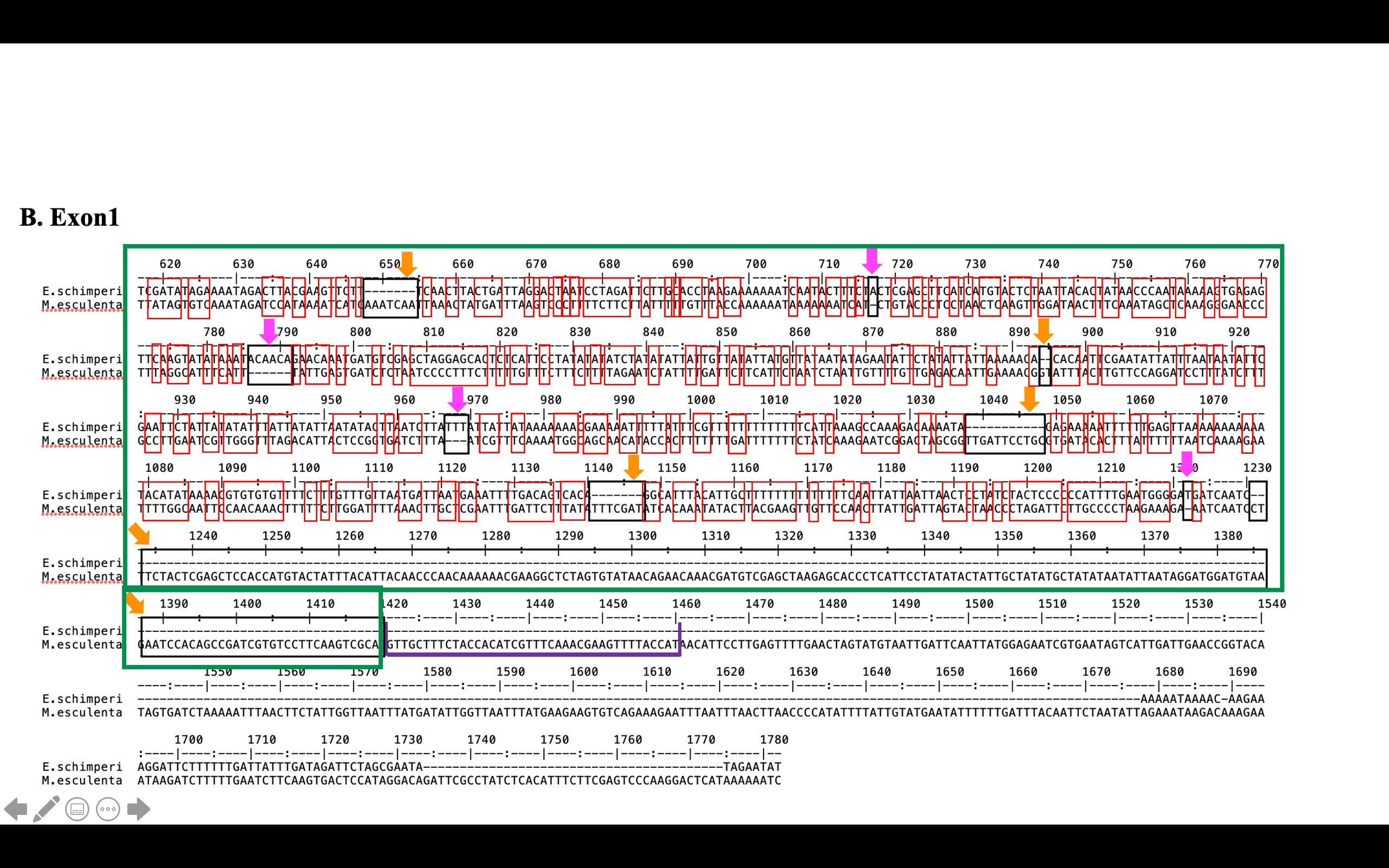


**Figure S2.** Alignment of pseudogene of *rps16* of *Euphorbia schimperi* with intact *rps16* of *Manihot esculenta*. **A.** Exon 2 (purple line) of *E. schimperi* alignment showing frameshift mutation of 5 bp deletion (orange arrow), 10 bp of insertion (pink arrow) and 27 bp substitution (red rectangle). **B**. Exon 1(purple line) alignment showing complete loss due to the deletion of 250 bp (black dash), insertion of 11 bp (pink arrow), and 338 bp substitutions (red rectangle) in intron (green rectangle). Exons of *rps16* of *Manihot* are underlined in purple. Red rectangles show bases that differ between *Manihot* and *Euphorbia*.


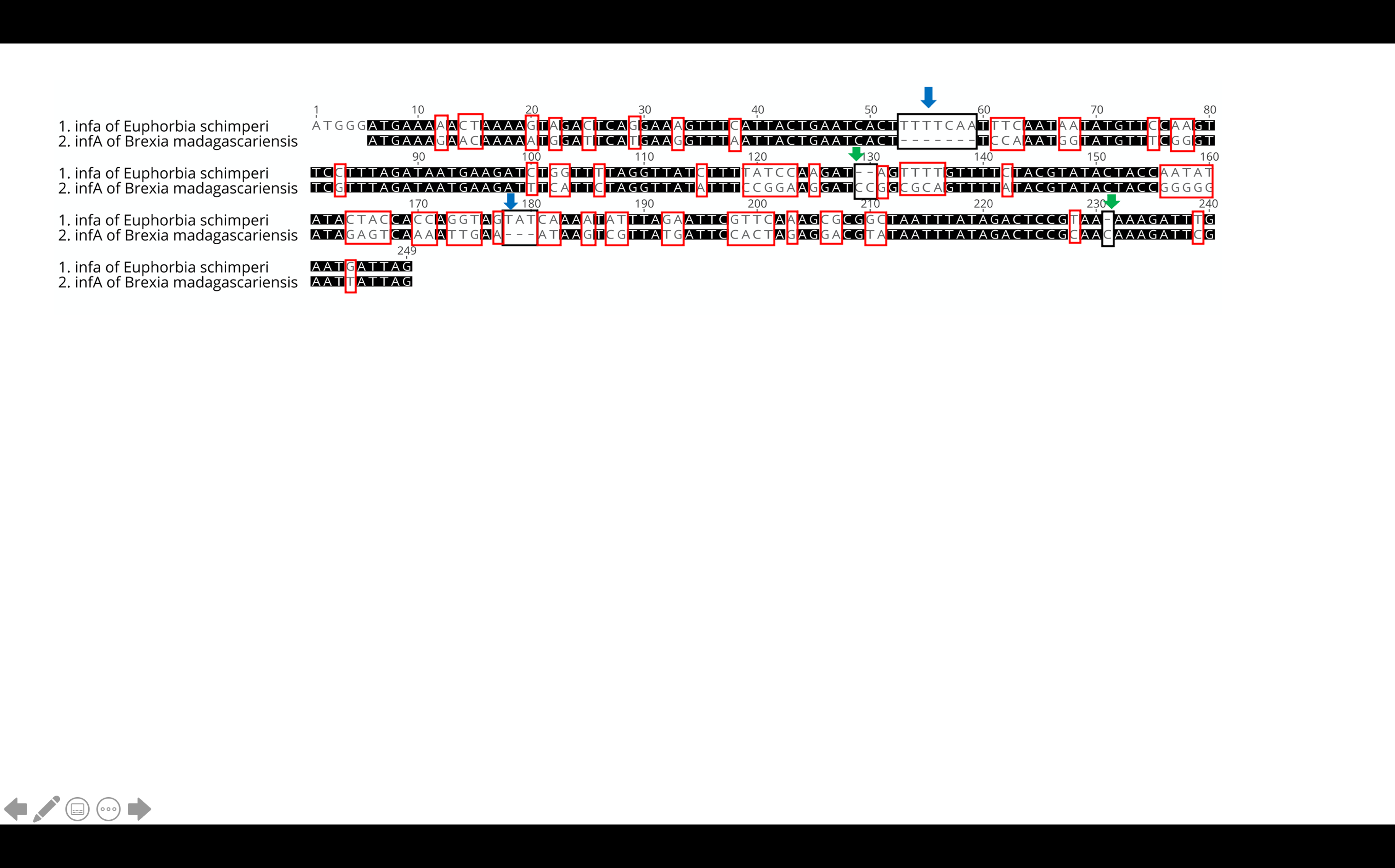


**Figure S3**. Alignment of the pseudogene of *infA* of *Euphorbia schimperi* with the intact *infA* of *Brexia madagascariensis* (AF347629) showing a 3-bp deletion (green arrow), 10 bp insertion (blue arrow), and 69 nucleotide substitutions (red rectangle) causing a frameshift. Red rectangles show bases that differ between *Euphorbia* and *Brexia*.


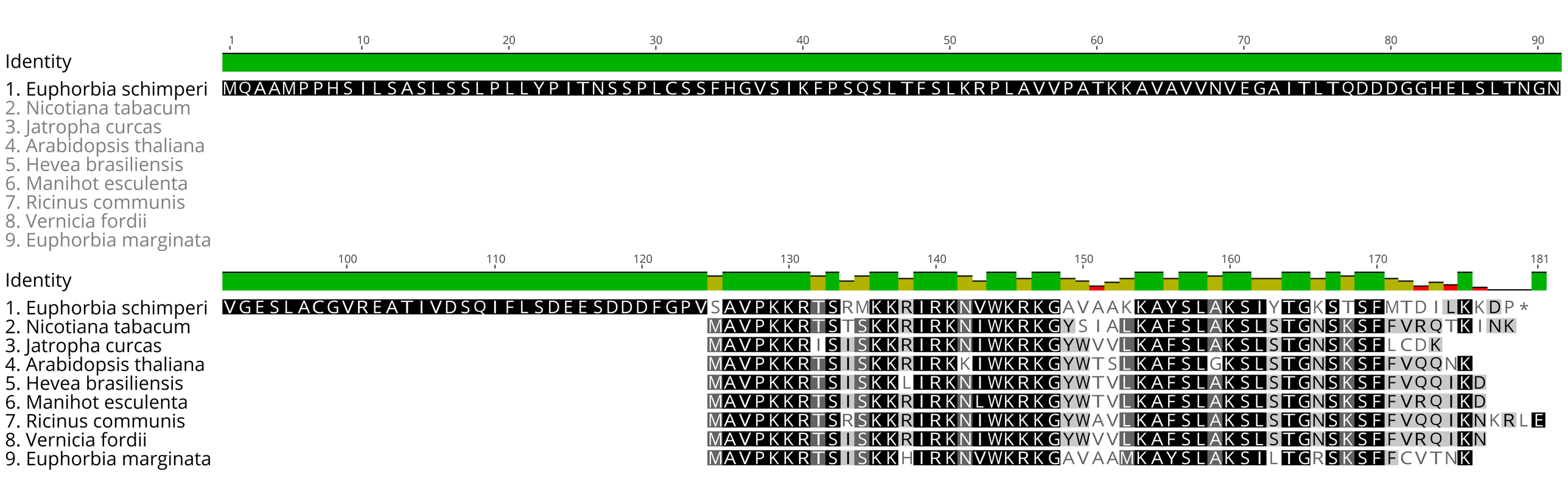


TP

**Figure 4**: Multiple alignments of nuclear **RPL32** of ***E.schimperi*** and **plastid** **RPL32** of other Euphorbiaceae (*E. marginata, Jatropha curcas, Hevea brasiliensis, Manihot esculenta, Ricinus communis, Vernicia fordii), Arabidopsis thaliana* and *Nicotiana tabacum.* Blue boxes indicate plastid transit peptide (TP) predicted using TargetP. Red box indicates a conserved domain of RPL32.

**ribosomal protein L32**
