# Supplementary material for: The evolutionary fate of rpl32 and rps16 losses in the Euphorbia schimperi (Euphorbiaceae) plastome
Source: Sci Rep. 2021 Apr 2;11:7466. doi: 10.1038/s41598-021-86820-z (PMC8018952; doi:10.1038/s41598-021-86820-z)
Supplement: Supplementary file 2 — Supplementary Tables. [file 41598_2021_86820_MOESM2_ESM.docx]

**The evolutionary fate of *rpl32* and *rps16* losses in the *Euphorbia schimperi* (Euphorbiaceae) plastome**

**Aldanah A. Alqahtani^1,2*^ and Robert K. Jansen^1,3^**

^1^Department of Integrative Biology, University of Texas at Austin, TX, 78712, USA

^2^Department of Biology, Prince Sattam Bin Abdulaziz University, Al-Kharj 11942, Saudi Arabia

^3^Centre of Excellence in Bionanoscience Research, Department of Biological Sciences, Faculty of Science, King Abdulaziz University, Jeddah 21589, Saudi Arabia

^*^Corresponding author

Aldanah A. Alqahtani

[aldanaha2016@utexas.edu](mailto:aldanaha2016@utexas.edu)

[Ald.alqahtani@psau.edu.sa](mailto:Ald.alqahtani@psau.edu.sa)

**Table S1**: Statistics of translated transcriptome assembly.

| Total length of sequence | 37,478,499 aa |
| --- | --- |
| Total number of contigs | 133,847 |
| N25 | 533 aa |
| N50 | 349 aa |
| N75 | 211 aa |
| Max contig length | 4,649 aa |
| Mean contig length | 280 aa |
| Total GC count | 3,062,484 aa |
| GC% | 8.17 % |

**Table S2**: List of 12 Families of Malpighiales and two outgroup species from Fabales used in phylogenetic analyses.

| Family | Species | NCBI Accession number |
| --- | --- | --- |
| Euphorbiaceae | *Croton tiglium* | NC_040113.1 |
| Euphorbiaceae | *Deutzianthus tonkinensis* | NC_041102.1 |
| Euphorbiaceae | *Euphorbia esula* | NC_033910.1 |
| Euphorbiaceae | *Euphorbia kansui* | MH392274.1 |
| Euphorbiaceae | *Euphorbia schimperi* | MT900567.1 |
| Euphorbiaceae | *Euphorbia larica* | MN646683.1 |
| Euphorbiaceae | *Euphorbia smithii* | MN646684.1 |
| Euphorbiaceae | *Euphorbia tirucalli* | NC_042193.1 |
| Euphorbiaceae | *Hevea brasiliensis* | NC_015308.1 |
| Euphorbiaceae | *Hevea camargoana* | MN781109.1 |
| Euphorbiaceae | *Jatropha curcas* | NC_012224.1 |
| Euphorbiaceae | *Manihot esculenta* | NC_010433.1 |
| Euphorbiaceae | *Ricinus communis* | NC_016736.1 |
| Euphorbiaceae | *Vernicia fordii* | NC_034803.1 |
| Euphorbiaceae | *Mallotus peltatus* | NC_047284.1 |
| Malpighiaceae | *Banisteriopsis caapi* | NC_037945.1 |
| Malpighiaceae | *Byrsonima coccolobifolia* | NC_037191.1 |
| Passifloraceae | *Passiflora edulis* | NC_034285.1 |
| Passifloraceae | *Passiflora cincinnata* | NC_037690.1 |
| Passifloraceae | *Passiflora contracta* | NC_043818.1 |
| Passifloraceae | *Passiflora pittieri* | NC_038125.1 |
| Salicaceae | *Populus euphratica* | NC_024747.1 |
| Salicaceae | *Populus alba* | NC_008235.1 |
| Salicaceae | *Bennettiodendron brevipes* | MK046729.1 |
| Violaceae | *Viola*  *seoulensis* | NC_026986.1 |
| Violaceae | *Viola mirabilis* | NC_041582.1 |
| Chrysobalanaceae | *Acioa guianensis* | NC_030534.1 |
| Chrysobalanaceae | *Afrolicania elaeosperma* | NC_030544.1 |
| Linaceae | *Linum usitatissimum* | NC_036356.1 |
| Erythroxylaceae | *Erythroxylum novogranatense* | NC_030601.1 |
| Clusiaceae | *Garcinia mangostana* | NC_036341.1 |
| Clusiaceae | *Garcinia gummi-gutta* | NC_047250.1 |
| Phyllanthaceae | *Glochidion chodoense* | NC_ 042906.1 |
| Irvingiaceae | *Klainedoxa gabonensis* | NC_044475.1 |
| Rhizophoraceae | *Rhizophora stylosa* | NC_042819.1 |
| Fabaceae | *Glycine max* | NC_007942.1 |
| Fabaceae | *Pisum sativum* | NC_014057.1 |

**Table S3.** List of 45 plastid encoded protein-coding genes used for phylogenetic analysis.

| Gene category | Genes |
| --- | --- |
| Photosystem I | *psaA, psaB, psaC, psaI, psaJ* |
| Photosystem II | *psbA, psbB, psbC, psbD, psbE, psbF, psbH, psbI, psbJ, psbK, psbL, psbM, psbN, psbT, psbZ.* |
| Cytochrome | *petA, petG,petL, petN* |
| ATP synthase | *atpA, atpB, atpE, atpH, atpI* |
| Ribosomal protein (large subunit) | *rpl33, rpl36* |
| Ribosomal protein (small subunit) | *rps2, rps3, rps4* |
| RNA polymerase | *rpoA, rpoB* |
| ATP dependent protease | *clpP* |
| Subunits of NADH-dehydrogenase | *ndhA, ndhB, ndhC, ndhH, ndhI, ndhJ, ndhK.* |
| Maturase | *matK* |

**Table S4**: List of 65 plastid-encoded *rpl32* genes and six nuclear encoded *rpl32* genes among angiosperms used for phylogenetic analyses.

| Groups | Order | Family | Species | NCBI accession numbers | Gene ID |
| --- | --- | --- | --- | --- | --- |
| Basal Angiosperms | Amborellales | Amborellaceae | *Amborella trichopoda* | NC_005086.1 | 2546583 |
| Basal Angiosperms | Austrobaileyales | Schisandraceae | *Illicium oligandrum* | NC_009600.1 | 5236718 |
| Basal Angiosperms | Chloranthales | Chloranthaceae | *Chloranthus spicatus* | NC_009598.1 | 5236496 |
| Basal Angiosperms | Nymphaeales | Nymphaeaceae | *Nuphar advena* | NC_008788.1 | 4699586 |
| Basal Angiosperms | Nymphaeales | Nymphaeaceae | *Nymphaea alba* | NC_006050.1 | 2896181 |
| Magnoliids | Canellales | Winteraceae | *Drimys granadensis* | NC_008456.1 | 4363555 |
| Magnoliids | Laurales | Calycanthaceae | *Calycanthus floridus* | NC_004993.1 | 2598028 |
| Magnoliids | Magnoliales | Magnoliaceae | *Liriodendron tulipifera* | NC_008326.1 | 4266681 |
| Magnoliids | Piperales | Piperaceae | *Piper coenoclatum* | NC_008457.1 | 4363679 |
| Monocots | Acorales | Acoraceae | *Acorus americanus* | NC_010093.1 | 5777705 |
| Monocots | Arecales | Arecaceae | *Elaeis guineensis* | NC_017602.1 | 12079485 |
| Monocots | Dioscoreales | Dioscoreaceae | *Dioscorea elephantipes* | NC_009601.1 | 5236576 |
| Monocots | Poales | Poaceae | *Oryza sativa* | NC_001320.1 | 3131486 |
| Monocots | Poales | Poaceae | *Sorghum bicolor* | NC_008602.1 | 4549149 |
| Monocots | Poales | Poaceae | *Triticum aestivum* | NC_002762.1 | 803150 |
| Monocots | Poales | Poaceae | *Zea mays* | NC_001666.1 | 845221 |
| Monocots | Zingiberales | Musaceae | *Musa textilis* | NC_022926.1 | 17728024 |
| Monocots | Alismatales | Lemnaceae | *Lemna minor* | NC_010109.1 | 5787533 |
| Eudicots | Apiales | Apiaceae | *Daucus carota* | NC_008325.1 | 4266807 |
| Eudicots | Apiales | Araliaceae | *Panax schinseng* | NC_006290 .1 | 3021562 |
| Eudicots | Asterales | Asteraceae | *Helianthus annuus* | NC_007977.1 | 4266807 |
| Eudicots | Asterales | Asteraceae | *Lactuca sativa* | NC_007578.1 | 3772819 |
| Eudicots | Brassicales | Brassicaceae | *Arabidopsis thaliana* | NC_000932.1 | 44704 |
| Eudicots | Brassicales | Brassicaceae | *Brassica rapa* | NC_015139.1 | 41704150 |
| Eudicots | Buxales | Buxaceae | *Buxus microphylla* | NC_009599.1 | 5236947 |
| Eudicots | Caryophyllales | Amaranthaceae | *Spinacia oleracea* | NC_002202.1 | 2715628 |
| Eudicots | Fabales | Fabaceae | *Glycine max* | NC_007942.1 | 3989364 |
| Eudicots | Fabales | Fabaceae | *Lotus corniculatus* | NC_002694.1 | 802916 |
| Eudicots | Fabales | Fabaceae | *Medicago truncatula* | NC_003119.1 | 5333097 |
| Eudicots | Gentianales | Rubiaceae | *Coffea arabica* | NC_008535.1 | 4421848 |
| Eudicots | Lamiales | Oleaceae | *Jasminum nudiflorum* | NC_008407.1 | 4319829 |
| Eudicots | Malvales | Malvaceae | *Gossypium hirsutum* | NC_007944.1 | 3989244 |
| Eudicots | Myrtales | Myrtaceae | *Eucalyptus globulus* | NC_008115.1 | 4108378 |
| Eudicots | Myrtales | Onagraceae | *Oenothera elata* | NC_002693.1 | 802766 |
| Eudicots | Proteales | Platanaceae | *Platanus occidentalis* | NC_008335.1 | 4271351 |
| Eudicots | Ranunculales | Berberidaceae | *Nandina domestica* | NC_008336.1 | 4271646 |
| Eudicots | Ranunculales | Ranunculaceae | *Ranunculus macranthus* | NC_008796.1 | 4712100 |
| Eudicots | Ranunculales | Ranunculaceae | *Megaleranthis saniculifolia* | NC_012615.1 | 7802835 |
| Eudicots | Rosales | Moraceae | *Morus indica* | NC_008359.1 | 4290604 |
| Eudicots | Sapindales | Rutaceae | *Citrus sinensis* | NC_008334.1 | 4271111 |
| Eudicots | Solanales | Solanaceae | *Atropa belladonna* | NC_004561.1 | 806572 |
| Eudicots | Solanales | Convolvulaceae | *Ipomoea purpurea* | NC_009808.1 | 5601298 |
| Eudicots | Solanales | Solanaceae | *Nicotiana tabacum* | NC_001879.1 | 800466 |
| Eudicots | Solanales | Solanaceae | *Solanum tuberosum* | NC_008096.1 | 4099902 |
| Eudicots | Cucurbitales | Cucurbitaceae | *Cucumis sativus* | NC_007144.1 | 3429298 |
| Eudicots | Malpighiales | Euphorbiaceae | *Manihot esculenta* | NC_010433.1 | 5999996 |
| Eudicots | Malpighiales | Euphorbiaceae | *Jatropha curcas* | NC_012224.1 | 7564794 |
| Eudicots | Malpighiales | Euphorbiaceae | *Vernicia fordii* | NC_034803.1 | 32891949 |
| Eudicots | Malpighiales | Euphorbiaceae | *Ricinus communis* | NC_016736.1 | 11542379 |
| Eudicots | Malpighiales | Euphorbiaceae | *Hevea brasiliensis* | NC_015308.1 | 10351950 |
| Eudicots | Malpighiales | Euphorbiaceae | *Hevea camargoana* | MN781109.1 | - |
| Eudicots | Malpighiales | Euphorbiaceae | *Croton tiglium* | NC_040113.1 | 38570643 |
| Eudicots | Malpighiales | Euphorbiaceae | Deutzianthus tonkinensis | NC_041102.1 | 39332492 |
| Eudicots | Malpighiales | Euphorbiaceae | *Mallotus peltatus* | NC_047284.1 | 54602857 |
| Eudicots | Malpighiales | Malipighiaceae | *Banisteriopsis caapi* | NC_037945.1 | 36953879 |
| Eudicots | Malpighiales | Malipighiaceae | *Byrsonima coccolobifolia* | NC_037191.1 | 36276246 |
| Eudicots | Malpighiales | Passifloraceae | *Passiflora cincinnata* | NC_037690.1 | 36934597 |
| Eudicots | Malpighiales | Passifloraceae | *Passiflora edulis* | NC_034285.1 | 32228278 |
| Eudicots | Malpighiales | Chrysobalanaceae | *Acioa guianensis* | NC_030534.1 | 28265250 |
| Eudicots | Malpighiales | Chrysobalanaceae | *Afrolicania elaeosperma* | NC_030544.1 | 28255866 |
| Eudicots | Malpighiales | Linaceae | *Linum usitatissimum* | NC_036356.1 | 35093902 |
| Eudicots | Malpighiales | Phyllanthaceae | *Glochidion chodoense* | NC_ 042906.1 | 40509272 |
| Eudicots | Malpighiales | Irvingiaceae | *Klainedoxa gabonensis* | NC_044475.1 | 41662131 |
| Eudicots | Caryophyllales | Caryophyllaceae | *Silene latifolia* | NC_016730.1 | 11541131 |
| Eudicots | Vitales | Vitaceae | *Vitis vinifera* | NC_007957.1 | 4025036 |
| Eudicots | Malpighiales | Passifloracea | *Passiflora tenuiloba* | - | MT259560.1 |
| Eudicots | Malpighiales | Euphorbiaceae | *Euphorbia schimperi* | - | MT913562.1 |
| Eudicots | Malpighiales | Salicaceae | *Populus alba* | - | AB302216.1 |
| Eudicots | Malpighiales | Rhizophoraceae | *Bruguiera gymnorrhiza* | - | AM711843.1 |
| Eudicots | Ranunculales | Ranunculaceae | *Thalictrum coreanum* | Online Data  Thalictrum data |  |
| Eudicots | Ranunculales | Ranunculaceae | *Aquilegia caerulea* | Online Data  Thalictrum data |  |

**Table S5**: List of 55 plastid-encoded *rps16* genes and 8 nuclear encoded *rps16* genes among angiosperms used for phylogenetic analyses.

| Groups | Order | Family | Species | NCBI accession numbers | Gene ID |
| --- | --- | --- | --- | --- | --- |
| Basal Angiosperms | Amborellales | Amborellaceae | *Amborella trichopoda* | NC_005086.1 | 2546610 |
| Basal Angiosperms | Austrobaileyales | Schisandraceae | *Illicium oligandrum* | NC_009600.1 | 5236697 |
| Basal Angiosperms | Chloranthales | Chloranthaceae | *Chloranthus spicatus* | NC_009598.1 | 5236444 |
| Basal Angiosperms | Nymphaeales | Nymphaeaceae | *Nuphar advena* | NC_008788.1 | 4699643 |
| Basal Angiosperms | Nymphaeales | Nymphaeaceae | *Nymphaea alba* | NC_006050.1 | 2896204 |
| Magnoliids | Canellales | Winteraceae | *Drimys granadensis* | NC_008456.1 | 4363626 |
| Magnoliids | Laurales | Calycanthaceae | *Calycanthus floridus* | NC_004993.1 | 2598040 |
| Magnoliids | Magnoliales | Magnoliaceae | *Liriodendron tulipifera* | NC_008326.1 | 4266583 |
| Magnoliids | Piperales | Piperaceae | *Piper coenoclatum* | NC_008457.1 | 4363763 |
| Monocots | Acorales | Acoraceae | *Acorus americanus* | NC_010093.1 |  |
| Monocots | Arecales | Arecaceae | *Elaeis guineensis* | NC_017602.1 | 12079506 |
| Monocots | Poales | Poaceae | *Oryza sativa* | NC_001320.1 | 3131439 |
| Monocots | Poales | Poaceae | *Sorghum bicolor* | NC_008602.1 | 4549140 |
| Monocots | Poales | Poaceae | *Triticum aestivum* | NC_002762.1 | 803100 |
| Monocots | Poales | Poaceae | *Zea mays* | NC_001666.1 | 845232 |
| Monocots | Zingiberales | Musaceae | *Musa textilis* | NC_022926.1 |  |
| Monocots | Alismatales | Lemnaceae | *Lemna minor* | NC_010109.1 | 5787601 |
| Eudicots | Apiales | Apiaceae | *Daucus carota* | NC_008325.1 | 4266704 |
| Eudicots | Apiales | Araliaceae | *Panax schinseng* | NC_006290 .1 | 23454007 |
| Eudicots | Asterales | Asteraceae | *Helianthus annuus* | NC_007977.1 | 4055640 |
| Eudicots | Asterales | Asteraceae | *Lactuca sativa* | NC_007578.1 |  |
| Eudicots | Brassicales | Brassicaceae | *Arabidopsis thaliana* | NC_000932.1 | 844798 |
| Eudicots | Brassicales | Brassicaceae | *Brassica rapa* | NC_015139.1 | 41704073 |
| Eudicots | Buxales | Buxaceae | *Buxus microphylla* | NC_009599.1 | 5236842 |
| Eudicots | Caryophyllales | Amaranthaceae | *Spinacia oleracea* | NC_002202.1 | 2715639 |
| Eudicots | Fabales | Fabaceae | *Glycine max* | NC_007942.1 | 3989305 |
| Eudicots | Fabales | Fabaceae | *Lotus corniculatus* | NC_002694.1 | 802894 |
| Eudicots | Gentianales | Rubiaceae | *Coffea arabica* | NC_008535.1 | 4421804 |
| Eudicots | Lamiales | Oleaceae | *Jasminum nudiflorum* | NC_008407.1 | 4319777 |
| Eudicots | Malvales | Malvaceae | *Gossypium hirsutum* | NC_007944.1 | 3989183 |
| Eudicots | Myrtales | Myrtaceae | *Eucalyptus globulus* | NC_008115.1 | 4108470 |
| Eudicots | Myrtales | Onagraceae | *Oenothera elata* | NC_002693.1 | 802811 |
| Eudicots | Proteales | Platanaceae | *Platanus occidentalis* | NC_008335.1 | 4271344 |
| Eudicots | Malpighiales | Clusiaceae | *Garcinia mangostana* | NC_036341.1 | 35092738 |
| Eudicots | Malpighiales | Clusiaceae | *Garcinia gummi-gutta* | NC_047250.1 | 54592019 |
| Eudicots | Ranunculales | Berberidaceae | *Nandina domestica* | NC_008336.1 | 4271618 |
| Eudicots | Ranunculales | Ranunculaceae | *Ranunculus macranthus* | NC_008796.1 | 4712138 |
| Eudicots | Ranunculales | Ranunculaceae | *Megaleranthis saniculifolia* | NC_012615.1 | 7802737 |
| Eudicots | Ranunculales | Ranunculaceae | *Thalictrum thalictroides* | NC_039433.1 | 38091577 |
| Eudicots | Ranunculales | Ranunculaceae | *Aquilegia caerulea* | NC_041528.1 | 39707697 |
| Eudicots | Rosales | Moraceae | *Morus indica* | NC_008359.1 | 4290656 |
| Eudicots | Sapindales | Rutaceae | *Citrus sinensis* | NC_008334.1 | 4271244 |
| Eudicots | Solanales | Solanaceae | *Atropa belladonna* | NC_004561.1 | 806549 |
| Eudicots | Solanales | Convolvulaceae | *Ipomoea purpurea* | NC_009808.1 | 5601319 |
| Eudicots | Solanales | Solanaceae | *Nicotiana tabacum* | NC_001879.1 | 800493 |
| Eudicots | Solanales | Solanaceae | *Solanum tuberosum* | NC_008096.1 | 4099950 |
| Eudicots | Cucurbitales | Cucurbitaceae | *Cucumis sativus* | NC_007144.1 | 3429310 |
| Eudicots | Malpighiales | Euphorbiaceae | *Manihot esculenta* | NC_010433.1 | 6000059 |
| Eudicots | Malpighiales | Euphorbiaceae | *Ricinus communis* | NC_016736.1 | 11542414 |
| Eudicots | Malpighiales | Euphorbiaceae | *Hevea brasiliensis* | NC_015308.1 | 10351887 |
| Eudicots | Malpighiales | Euphorbiaceae | *Hevea camargoana* | MN781109.1 | - |
| Eudicots | Malpighiales | Euphorbiaceae | *Croton tiglium* | NC_040113.1 | 38570550 |
| Eudicots | Malpighiales | Phyllanthaceae | *Glochidion chodoense* | NC_ 042906.1 | 40509167 |
| Eudicots | Caryophyllales | Caryophyllaceae | *Silene latifolia* | NC_016730.1 | 11541164 |
| Eudicots | Vitales | Vitaceae | *Vitis vinifera* | NC_007957.1 | 4025122 |
| Eudicots | Malpighiales | Passifloracea | *Passiflora tenuiloba* | - | QKY65187.1  QKY65184.1 |
| Eudicots | Malpighiales | Passifloracea | *Passiflora pittieri* |  | QKY65183.1  QKY65185.1 |
| Eudicots | Malpighiales | Euphorbiaceae | *Euphorbia schimperi* | - | (MT897997) (MT897998) |
| Eudicots | Malpighiales | Salicaceae | *Populus alba* | - | BAG49074.1  BAG49075.1 |
